# The Role of Neuropeptide Y in Cardiovascular Health and Disease

**DOI:** 10.3389/fphys.2018.01281

**Published:** 2018-09-19

**Authors:** Cheryl M. J. Tan, Peregrine Green, Nidi Tapoulal, Adam J. Lewandowski, Paul Leeson, Neil Herring

**Affiliations:** ^1^Oxford Cardiovascular Clinical Research Facility, Division of Cardiovascular Medicine, Radcliffe Department of Medicine, University of Oxford, Oxford, United Kingdom; ^2^Department of Physiology, Anatomy and Genetics, Burdon Sanderson Cardiac Science Centre, University of Oxford, Oxford, United Kingdom

**Keywords:** Neuropeptide Y (NPY), sympathetic nervous system, co-transmission, cardiovascular disease, hypertension, myocardial infarction, heart failure, arrhythmia

## Abstract

Neuropeptide Y (NPY) is an abundant sympathetic co-transmitter, widely found in the central and peripheral nervous systems and with diverse roles in multiple physiological processes. In the cardiovascular system it is found in neurons supplying the vasculature, cardiomyocytes and endocardium, and is involved in physiological processes including vasoconstriction, cardiac remodeling, and angiogenesis. It is increasingly also implicated in cardiovascular disease pathogenesis, including hypertension, atherosclerosis, ischemia/infarction, arrhythmia, and heart failure. This review will focus on the physiological and pathogenic role of NPY in the cardiovascular system. After summarizing the NPY receptors which predominantly mediate cardiovascular actions, along with their signaling pathways, individual disease processes will be considered. A thorough understanding of these roles may allow therapeutic targeting of NPY and its receptors.

## Introduction

Neuropeptide Y (NPY) is a highly conserved peptide, which is abundantly distributed in the central and peripheral nervous system and was first discovered in porcine brain (Tatemoto et al., 1982; Pedrazzini et al., 2003). NPY is mainly found in post-ganglionic sympathetic neurons, from which it is released simultaneously with norepinephrine (NE) (Lundberg et al., 1990; Larhammar, 1996) in response to sympathetic stimulation, functioning as a “co-transmitter.” Geoffrey Burnstock was the first to suggest that neurons release more than one neurotransmitter (Burnstock, 1976). Henry Dale's principle had to be revised to state that “at all the axonal branches of a neuron, there is liberation of the same transmitter substance *or substances*” to incorporate Burnstock's theory that nerves of the same class utilize more than one neurotransmitter (Eccles, 1976). Sympathetic nerves contain co-transmitters such as adenosine triphosphate (ATP) and galanin in addition to NPY, although significant co-transmitter release generally occurs only on high-level neuronal stimulation (Lundberg et al., 1986). Unlike ATP which is rapidly metabolized (Burnstock, 1976), NPY and galanin are slowly diffusing molecules with a much longer half-life and duration of action than classical neurotransmitters (Lundberg, 1996).

NPY is a prominent player in a variety of physiological functions, including the regulation of mood, cardiovascular and immune homeostasis, vasomotion, angiogenesis, cardiac remodeling, appetite, gastrointestinal motility, neuroendocrine axis, sympathetic and vagal transmission (Hellstorm et al., 1985; Yang and Levy, 1992; Grundemar and Hakanson, 1993; Wan and Lau, 1995; Michel et al., 1998). NPY is the most abundant neuropeptide in the heart (Gu et al., 1983), and is present in post-ganglionic sympathetic neurons supplying the vasculature, endocardium and cardiomyocytes, as well as in intracardiac ganglia and parasympathetic neurons (Mcdermott and Bell, 2007). However, in addition to its important role in normal physiological control mechanisms, it is also increasingly implicated in the pathophysiology of a number of cardiovascular disease processes. In humans and animals, elevated plasma NPY levels were observed in several stress conditions including exercise, hypoxia, cold exposure, tissue injury, ischemia, and hemorrhagic shock (Pernow et al., 1987), and dysregulation of NPY has been implicated in the pathophysiology of metabolic disorders (Dvorakova et al., 2014). Furthermore, increased plasma NPY levels are also found in pathological conditions with sympathetic hyperactivity such as hypertension, left-ventricle hypertrophy, and heart failure (Zukowska-Grojec et al., 1991, 1998b; Dvorakova et al., 2014). These roles raise the potential for the therapeutic targeting of NPY receptors in novel therapies for cardiovascular disease.

This review will summarize the physiological functions of NPY, its receptors and their associated signaling pathways in the cardiovascular system, before focussing on the current understanding of the role of NPY in physiological cardiovascular regulation and different cardiovascular disease processes.

## The NPY family of peptides

### Neuropeptide Y

NPY is a 36-residue peptide amide (Tatemoto, 1982) synthesized and released by sympathetic nerves and the adrenal medulla (Pedrazzini et al., 2003; Zukowska et al., 2003b). The biosynthesis of functional NPY is achieved via the cleavage of a precursor (pre-pro-NPY) to generate NPY_1−36_, that is further truncated by the enzyme dipeptidyl peptidase 4 (DPP-4) to produce NPY_3−36_ (Aizawa-Abe et al., 2000; Robich et al., 2010; Dvorakova et al., 2014). NPY modulates its functions on the cardiovascular, central, and peripheral systems via the activation of a G protein-coupled Y-family of receptors (GPCR; Y1R,Y2R, Y3R, Y4R, Y5R, and γ6R) (Li et al., 2011; Troke et al., 2013). Similar to peptide YY (PYY) and pancreatic polypeptide (PP), NPY is one of the “PP fold” peptides (Pedrazzini et al., 2003). Despite structural similarities, each “PP fold” peptides have specific functionalities (Troke et al., 2013).

### Peptide YY (PYY) and pancreatic polypeptide (PP)

Released primarily in the gastrointestinal (GI) system by enteroendocrine cells, PYY influences appetite, GI motility, water, and electrolyte absorption (Brothers and Wahlestedt, 2010; Troke et al., 2013; Torang et al., 2015; Saraf et al., 2016). The most abundant PPY, the anorexigenic PPY_3−36_, is a truncated version of PPY (PPY_1−36_) post-DPP-4 cleavage (Torang et al., 2015). Similarly, PPY mediates its activity via the activation of GPCR; PYY_1−36_ binds to most Y family receptors (Y1R-Y5R) whereas, PYY_3−36_ is selective and has a higher affinity for the Y2R and Y5R (Brothers and Wahlestedt, 2010; Troke et al., 2013; Saraf et al., 2016). Upon food consumption, the pancreas stimulates the release of PP, triggering food absorption primarily via binding to Y4R (Saraf et al., 2016). Further consideration of PYY and PP is beyond the scope of this review.

## Overview of receptor subtypes and signaling in the cardiovascular system

Understanding of the molecular NPY receptor signaling pathways is crucial to facilitate therapeutic development (Pedrazzini et al., 2003). Among the six identified Y family GPCR, Y1R, Y2R, and Y5R are the main cardiovascular homeostasis regulators (Silva et al., 2002; Mcdermott and Bell, 2007). These receptors are expressed in the peripheral and central nervous system, including within the blood vessels, cardiomyocytes and enteroendocrine cells (Brothers and Wahlestedt, 2010; Troke et al., 2013). The receptors display some common and distinct functions, with different peptide preferences (Gehlert, 2004; Brothers and Wahlestedt, 2010; Troke et al., 2013) (Table 1).

**Table 1:** The characteristics of neuropeptide Y receptors.

**NPY receptor**	**Central and peripheral localisation**	**Peptide preference**	**Functional effect**	**Associated pathology**	**References**
Y1	Central: Cerebral cortex; brainstem and thalamus Peripheral: Vascular smooth muscle; immune cells; osteoblasts; heart and gastrointestinal system	NPY, [Leu^31^, Pro^34^] NPY, PYY > NPY_3−36_, PP	Food intake regulation; anxiolysis; regulation of neurotransmitter release; vasoconstriction; anti-depressant and bone metabolism	Cardiovascular morbidities including hypertension, cardiac hypertrophy, heart failure, ischemia and circadian disorders	(Larhammar, 1996; Herzog, 2003; Pedrazzini et al., 2003; Brothers and Wahlestedt, 2010; Troke et al., 2013)
Y2	Central: Hippocampus; brainstem and hypothalamus Peripheral: Autonomic nerves; immune cells endothelial cells; adipocytes; heart and gastrointestinal system	NPY, NPY_3−36_, NPY_13−36_, NPY_2−36_, PYY > [Leu^31^,Pro^34^]NPY, [Leu^31^,Pro^34^]PYY, PP	Neurotransmitter (glutamate) inhibition; learning and memory; inhibition of norepinephrine release; gastrointestinal motility; angiogenesis; blood pressure regulation and adipogenesis	Cardiovascular morbidities; circadian disorders; cancer; and intestinal disease.	(Balasubramaniam, 1997), (Pedrazzini et al., 2003; Gehlert, 2004; Abe et al., 2007; Brothers and Wahlestedt, 2010; Troke et al., 2013)
Y5	Central: Hippocampus; olfactory bulb; suprachiasmatic nucleus; arcuate nucleus Peripheral: Vascular smooth muscle; immune cells; heart and gastrointestinal system	NPY, PYY, [Leu^31^, Pro^34^] NPY, NPY_2−36_, NPY_3−36_ > PP	Food intake regulation; anxiolysis; antidepressant; angiogenesis	Cardiovascular disease and cancer	(Silva et al., 2002; Pedrazzini et al., 2003; Brothers and Wahlestedt, 2010; Hirsch and Zukowska, 2012)

**Figure 1:**
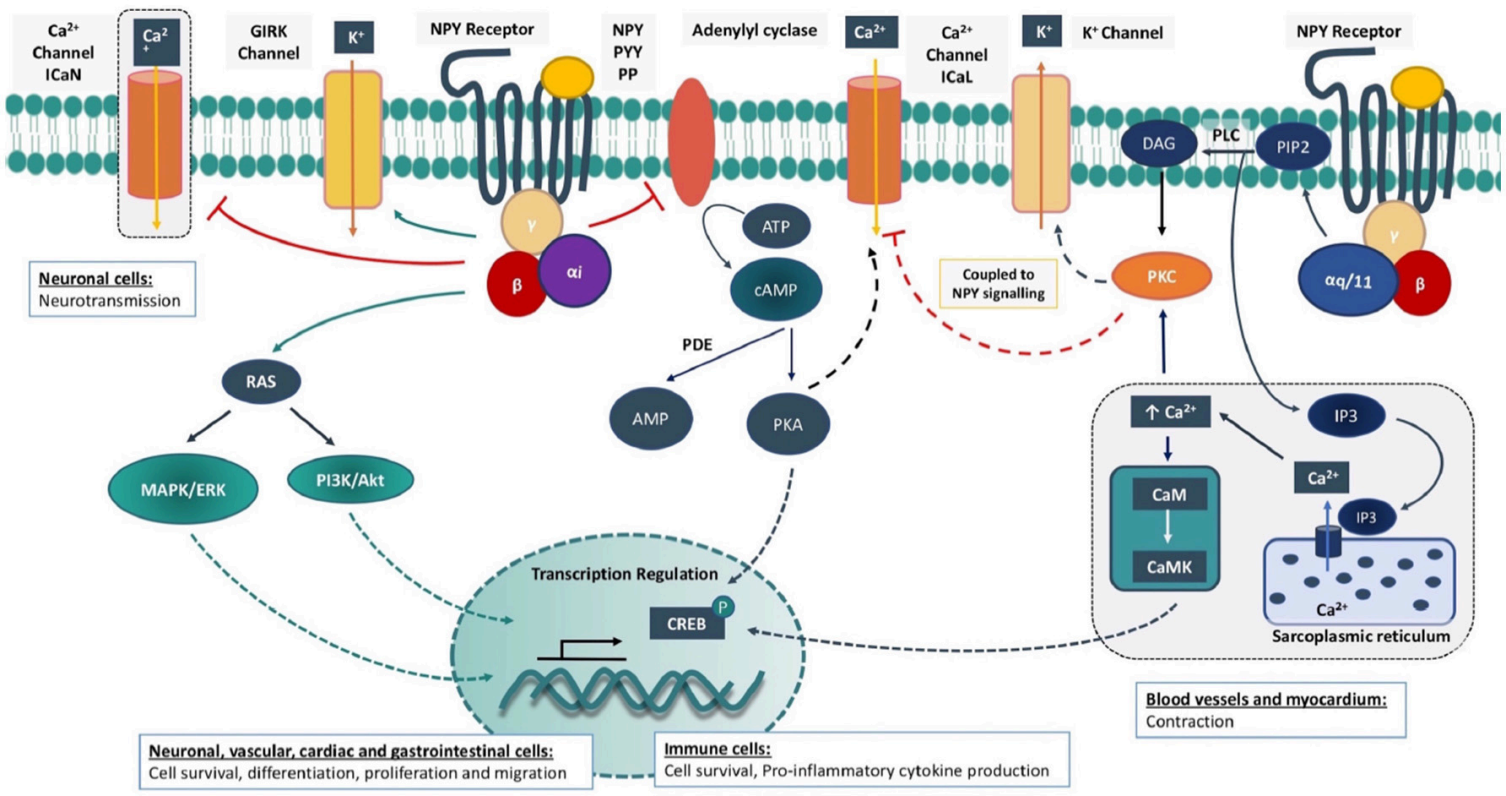
Intracellular signaling cascades for Neuropeptide Y receptors. Neuropeptide Y (NPY) receptor is a G-protein coupled receptor (GPCR) that is activated by specific peptides such as NPY, peptide YY (PYY) or pancreatic peptide (PP). The activated GPCR enhances the Gi signaling cascade where the G-protein complex inhibits adenylyl cyclase and cAMP/PKA stimulation of L-type Ca^2+^ current (ICaL). In neurons, direct G-protein coupling can also inhibit N-type Ca^2+^ current (ICaN) and neurotransmission. The G-protein complex can stimulate the inwardly rectifying potassium (GIRK) current. Mitogen-activated protein kinase/extracellular signal-regulated kinase (MAPK/ERK) and phosphatidylinositol-3-kinase/ protein kinase B (PI3K/Akt) signaling cascades are also activated. The Y1 receptor (Y1R) can also promote a Gq signaling cascade to stimulate Ca^2+^ release from the sarcoplasmic reticulum via inositol triphosphate (IP3). Elevation of intracellular Ca^2+^ activates Ca^2+^/ calmodulin-dependent kinase (CaMK) and protein kinase C (PKC). PKC activation is further enhanced via diacylglycerol (DAG), formed post-phosphatidylinositol 4,5-bisphosphate (PIP2) cleavage via phospholipase c (PLC). Initiation of these cellular pathways has a cell-dependent multitude of effects in the regulation of ion channels, calcium handling and transcription factors that hold the basis for the short and long term physiological effects of NPY. ATP, Adenosine 5′-triphosphate; cAMP, cyclic adenosine monophosphate; PDE, phosphodiesterase; AMP, adenosine monophosphate; PKA, protein kinase A; CREB, cAMP response element binding protein.

Molecularly, Y1R, Y2R, and Y5R are coupled to a G_α*i*_ subunit and have been shown to hinder the synthesis of cyclic adenosine monophosphate (cAMP) via adenylyl cyclase inhibition (Kassis et al., 1987; Motulsky and Michel, 1988; Ali et al., 2016), reducing protein kinase A (PKA) dependent stimulation of the L-type calcium (Ca^2+^) current (Bryant and Hart, 1996; Larhammar, 1996; Lee et al., 2003; Gehlert, 2004; Troke et al., 2013). There may also be direct G protein coupling to inhibit N-type Ca^2+^ channels (Hirning et al., 1990; Wiley et al., 1990) and stimulate inwardly rectifying potassium (K^+^) channels (GIRK) (Sun et al., 2001; Acuna-Goycolea et al., 2005). The Y1R can also couple to mobilization of intracellular Ca^2+^ stores depending on the cell type (Aakerlund et al., 1990; Herzog et al., 1992) via a G_q_-phospholipase C (PLC) dependent pathway (Del Puy Heredia et al., 2005). The IP_3_ dependent mobilization of intracellular Ca^2+^ can have a positive inotropic or vasoconstrictive effect, provoke contractile sensitisation, neurological functions such as learning and memory, immune regulation, lipid metabolism, and transcriptional regulation via the Ca^2+^-binding messenger calmodulin (CaM) (Troke et al., 2013; Lecat et al., 2015). The multi-functional effect of the Ca^2+^/CaM complex may be induced by the activation of CaM kinases such as Ca^2+^/CaM-dependent protein kinase II (CaMKII) and myosin light chain kinase (MLCK) (Leonard et al., 1999; Anderson, 2005). For instance, activation of CaMKII can influence ion channel activity, intracellular calcium handling and arrhythmogenesis, and can also induce the activation of neurotransmitter secretion, glycogen metabolism and transcriptional regulation (Anderson, 2005). On the other hand, MLCK performs a more specific action, phosphorylating the regulatory light chain of myosin (MLC_20_) to stimulate smooth muscle contraction (Tansey et al., 1994) (Figure 1). PLC signaling via diacylglycerol (DAG) and protein kinase C (PKC) can also inhibit and stimulate L-type Ca^2+^ current (Mccullough et al., 1998) and the transient outward K^+^ current (I_to_), respectively (Heredia et al., 2002). NPY signaling targeting Ca^2+^ and K^+^ current as well-intracellular calcium handling, is likely to explain its actions on neurotransmission, vascular reactivity, and cardiac excitability.

In addition, these receptors mediate the transactivation of insulin growth factor (IGF) receptor, the activation of mitogen-activated protein kinase/extracellular signal-regulated kinase (MAPK/ERK) and phosphatidylinositol-3-kinase (PI3K) signaling cascades that are important regulators of cellular growth, survival, proliferation, differentiation, and motility (Zhou et al., 2008; Lecat et al., 2015). Among the three main cardiac NPY receptors, Y5R phosphorylation is mediated by protein kinase C (PKC) to promote intracellular Ca^2+^ release (Herzog, 2003; Gehlert, 2004) (Figure 1).

### The neuropeptide Y1 receptor

Peripherally, Y1R modulates macrophage immunity, Ca^2+^ cycling, vasomotion, appetite, and anxiolysis (Gehlert, 2004; Jacques et al., 2006; Troke et al., 2013). The activation of Y1R within the cardiovascular system contributes to the sympathetic stimulation that accelerates NE-induced vasoconstriction (Troke et al., 2013; Saraf et al., 2016). These were further validated in studies that utilized a Y1R agonist and antagonist that induced direct vasoconstriction in kidneys and spleen and inhibited spontaneous vascular contraction, respectively (Saraf et al., 2016; Oki et al., 2017). Y1R-mediated vasoconstriction exhibits a gender-specific effect, as a more pronounced effect is observed in males rather than females due to the testosterone-induced NPY gene upregulation (Zukowska-Grojec et al., 1991). In addition, Y1R activation stimulates protein degradation, mitogenesis and migration of vascular smooth muscle cells (VSMCs) and endothelial cells (Movafagh et al., 2006; Li et al., 2011). In the heart, myocyte Y1R stimulation may exert a positive inotropic action through increased sarcoplasmic reticulum calcium release (Del Puy Heredia et al., 2005).

### The neuropeptide Y2 receptor

Peptide activation of Y2R can stimulate endothelial proliferation, adhesion and migration, thereby promoting angiogenesis (Wahlestedt and Reis, 1993; Gehlert, 2004; Troke et al., 2013). The NPY potent angiogenic property has been shown via the activation of Y2Rs in various conditions ranging from tumorigenesis and tissue ischemia, in part due to release of vascular endothelial growth factor (VEGF) (Lee et al., 2003; Li et al., 2011; Tilan et al., 2013). Furthermore, loss or inhibition of Y2Rs and DPP-4 severely impedes vascular angiogenesis in those circumstances (Tilan et al., 2013). These effects can also be confounded by age, as aged mice have compromised angiogenesis that is accompanied with a loss of Y2R and DPP-4 expression (Kitlinska et al., 2005). In addition, Y2R is involved in fine-tuning NE secretion in the periphery and myocardium at the presynaptic level (Zukowska et al., 2003b). The Y2R is also involved in the crosstalk between sympathetic and parasympathetic neurotransmission (Yang and Levy, 1992; Ilebekk et al., 2005; Herring et al., 2012). The exogenous NPY binds to Y2R that is present on the parasympathetic nerve terminals, to inhibit acetylcholine (ACh) release and vagal bradycardia in mammalian hearts, including human (Schwertfeger et al., 2004), canine (Ilebekk et al., 2005), and guinea pigs (Herring et al., 2008).

### The neuropeptide Y5 receptor

With slight structural alterations, Y5R comprises an additional 100 amino acid and extended third cytoplasmic loop as compared to other NPY receptors (Gehlert, 2004). The role of Y5R is elusive and has been shown to perform a synergistic function with not only Y1R to modulate vasomotion and mitogenesis of VSMCs (Zukowska-Grojec et al., 1998a; Nicholl et al., 2002; Herzog, 2003), but also with Y2R to promote vascular angiogenesis and arteriogenesis (Herzog, 2003; Li et al., 2003; Pons et al., 2003; Troke et al., 2013). A study conducted by Li et al. demonstrated the synergistic effects of Y2R and Y5R in ischemia revascularization in hindlimb ischemia of rats (Lee et al., 2003). In fact, Movafagh et al. revealed Y5R activity as an enhancer which markedly enriched endothelial cell proliferation, migration and tube formation alongside Y1R and Y2R (Movafagh et al., 2006).

## Neuropeptide Y as an important mediator of cardiovascular disease

**Figure 2:**
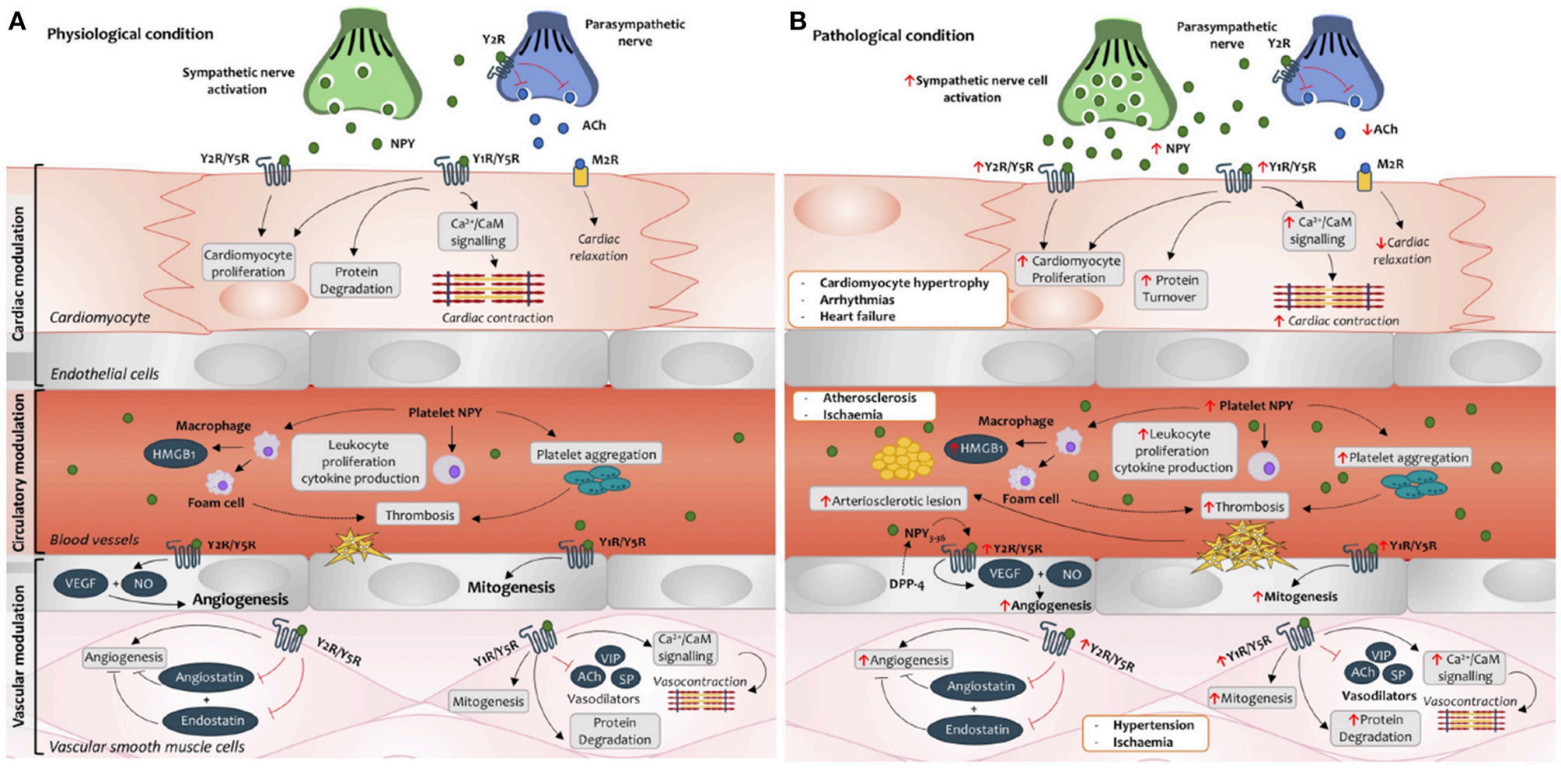
Neuropeptide Y signaling cascade in cardiovascular tissues during physiological **(A)** and pathological **(B)** conditions. **(A)** Under physiological conditions, autocrine and paracrine effects of NPY modulate cardiac contractibility, protein degradation and proliferation in the cardiomyocytes. Exogenous NPY expression via the cardiac sympathetic nerves can bind to the Y2 receptor (Y2R) on the parasympathetic nerve to inhibit acetylcholine (ACh) release and its binding to the muscarinic 2 receptor (M2R), limiting cardiac relaxation and bradycardia. Circulatory platelet NPY can regulate immune processes such as platelet aggregation, leukocyte activation and cytokine production. In the vessels, NPY activation can stimulate vasoconstriction, mitogenesis, and induce production of vascular endothelial growth factor (VEGF) and nitric oxide (NO) to promote angiogenesis. Furthermore, activation of NPY can inhibit the functional effects of anti-angiogenic factors (angiostatin and endostatin) and vasodilators such as substance P (SP), vasoactive intestinal peptide (VIP) and ACh. **(B)** Under pathological conditions, NPY production, receptor expression and dipeptidyl peptidase-4 (DPP-4) are increased in the cardiovascular tissues. These cause an imbalance of the sympatho-vagal interaction, cardiac contraction, cardiac remodeling, enhanced angiogenesis and proliferation and inflammation-induced endothelium dysfunction within the cardiac tissues. As such, altered NPY levels and activities underline the pathogenesis of various cardiovascular morbidities such as cardiac hypertrophy, arrhythmias, heart failure, arteriosclerosis, ischemia and hypertension. HMGB1, high mobility group protein B1.

Several studies have identified NPY as a functional cardiac regulator via direct and indirect cardiac nerve interactions (Zukowska-Grojec et al., 1998b). NPY displays a dual-opposing effect in cardiovascular tissues, including acting as a cardio-depressant, and a cardiac stressor (Pons et al., 2003). These contradictory effects of NPY are largely dependent on the pattern of the NPY receptor expression, ligand concentration, DPP-4 activity, and adrenergic activity (Pons et al., 2003; Kitlinska et al., 2005). The cardiac-related NPY receptors, Y1R, Y2R and Y5R have been commonly implicated in pathological states and have been suggested to be prominent players in the pathogenesis of cardiovascular diseases including hypertension, atherosclerosis, myocardial ischemia/infarction, diabetic, stress and hypertrophic cardiomyopathies, and heart failure (Zukowska-Grojec et al., 1991; Lee et al., 2003; Jacques and Abdel-Samad, 2007; Luo et al., 2015) (Figure 2).

### Neuropeptide Y and hypertension

Highly expressed in sympathetic nerve endings around the vasculature, NPY acts as a potent vasoconstrictor alongside NE (Dumont et al., 1992; Han et al., 1998). The circulating baseline plasma levels are relatively low in healthy individuals (Lunderberg et al., 1982; Renshaw and Hinson, 2001), but are significantly increased during conditions of sympathetic activation observed in stressful situations such as exercise (Lewis et al., 1993; Nickel et al., 2012), hypoxia (Kaijser et al., 1990), and cold (Han et al., 1998; Li et al., 2005). Plasma levels of NPY and NE are also increased in hypertensive patients and rats, suggesting enhanced sympathetic activity (Howe et al., 1986; Erlinge et al., 1994; Thulin and Erlinge, 1995; Velkoska et al., 2005). The increase in circulating NPY is observed and mediated by the peripheral nerves and adrenal medulla (Pedrazzini et al., 2003; Baltatzi et al., 2008). Elevated sympathetic activity at the level of the cardiac post-ganglionic neuron is also observed in the spontaneously hypertensive rat before the onset of hypertension (Li et al., 2012; Shanks et al., 2013) where it also gives rise to elevated NPY levels (Shanks et al., 2013). NPY can promote Ca^2+^/CaM mediated MLCK phosphorylation of MLC_20_ to initiate vasoconstriction (Dumont et al., 1992). In addition, via inhibiting adenylyl cyclase NPY impedes the action of several vasodilators including substance P, acetylcholine and vasoactive intestinal peptide (VIP) (Servin et al., 1989; Dumont et al., 1992).

More recently, elevated NPY levels have been observed in hypertensive pregnancies (preeclampsia) in humans (Paiva et al., 2016) and rodent models (Zhang et al., 2015a; Yi et al., 2018). Preeclampsia is a pregnancy complication that involves hypertension and vasculature remodeling (Lazdam et al., 2012). Zhang et al. underlined that pregnant hypertensive rats display elevated circulating NPY levels (Zhang et al., 2015a). An increase in circulating NPY promotes VSMC proliferation and migration through the activation of Y1R and Y5R *in vitro* and in vascular tissues via Y5R *in vivo* (Zhang et al., 2015a). Pathophysiological vasculature remodeling in hypertensive pregnancies is regulated through several signaling pathways, including signal transducer and activator of transcription 3 (STAT3), c-FOS, MAPK, and Ca^2+^ signaling via GPCR (Zhang et al., 2015a; Yi et al., 2018). As the physiological and molecular mechanisms of hypertensive pregnancy are highly complex (Davis et al., 2012), further observational and mechanistic studies will be required to validate the specific role of NPY on blood pressure regulation in this condition.

### Neuropeptide Y and atherosclerosis

Atherosclerosis is one of the leading cardiovascular diseases, with high global mortality rates. It involves complex molecular and cellular responses comprising of inflammation, proliferation, and matrix modification, following endothelial dysfunction which is a critical atherosclerosis-initiating factor (Jiang et al., 2017). Endothelial dysfunction enhances endothelium permeability and promotes leukocyte adhesion, lipid aggregation, macrophage-induced foam cell formation and platelet aggregation. It also modulates endothelial cell gene expression and VSMC proliferation and migration, thereby facilitating the formation of atherosclerotic plaque (Burnstock, 1976; Dzau et al., 2002; Li et al., 2003, 2011; Zukowska et al., 2003a; Zhu et al., 2016). Studies have highlighted the association of atherosclerotic burden and vulnerability with enhanced NPY activation in patients with arterial disease (Li et al., 2005, 2011). NPY interacts with receptors on endothelial cells, VSMCs, macrophages, and platelets (Li et al., 2011), (Racchi et al., 1997; Li et al., 2005). Under pathological conditions such as vascular endothelium injury, elevations in sympathetic glial-derived and platelet NPY and its receptors have been shown in mammalian (human and animal) studies (Burnstock, 1976; Ghersi et al., 2001; Li et al., 2003; Legein et al., 2013; Zhu et al., 2016; Jiang et al., 2017).

Synergistic activation of Y1R and Y5R in endothelial cells and VSMCs increases the intracellular Ca^2+^, PKC and MAPK activities that enhance NE-induced vasoconstriction, resulting in blood pressure elevation, local vascular spasm, stenosis and endothelial cell/VSMC retraction and discontinuity (Burnstock, 1976; Lundberg et al., 1986; Racchi et al., 1997; Aizawa-Abe et al., 2000; Ghersi et al., 2001; Li et al., 2005; Jacques and Abdel-Samad, 2007; Ruohonen et al., 2009). The activation of endothelial cell and VSMC Y1Rs and Y5Rs can also trigger cellular proliferation that contributes to the progression of intima thickening (Zukowska-Grojec et al., 1998a, Pons et al., 2003; Li et al., 2005). Increases in NPY and Y1R and Y5R are also associated with platelet aggregation and adhesion following thrombosis (Ruohonen et al., 2009). Zhou et al. showed that binding of NPY to Y1R on macrophages promotes the synthesis and production of pro-inflammatory high mobility group protein B1 (HMGB1) via a PKC/ERK dependent pathway, which can damage endothelium integrity (Zhou et al., 2013).

In rats, an angioplasty injury model of carotid arteries has demonstrated that NPY stimulates the formation of atherosclerotic plaque lesions, accompanied with increased Y1R and Y5R expression, proliferation of VSMC and neovascularization of plaque (Li et al., 2005; Ruohonen et al., 2009). These not only intensify plaque growth but also enhance the risk of plaque rupture and hemorrhage (Li et al., 2005; Ruohonen et al., 2009). The NPY-induced effects on atherosclerotic lesions can be abolished by a Y1R antagonist (Li et al., 2005). Activated Y2R on endothelial cells and VSMCs promote the secretion of NO and VEGF, inhibiting anti-angiogenic endostatin and angiostatin, to facilitate proliferation, migration, and angiogenesis (Erlinge et al., 1994; Kitlinska et al., 2005; Zhu et al., 2016). The ischemic and inflamed atherosclerotic tissue triggers an elevation in DPP-4 secretion within the endothelium to increase cleavage of NPY_1−36_ to NPY_3−36_ (Ghersi et al., 2001). The peptide NPY_3−36_ has a higher binding affinity for Y2R and Y5R, thereby shifting NPY activity toward a pro-angiogenic profile, further enhancing vascularization and lesion vulnerability (Li et al., 2011). Nonetheless, some studies have underlined the gender-specific effects of the positive relationship of NPY and atherosclerotic plaque neovascularization upregulation in males only, suggesting a potential androgen-dependent effect (Zukowska-Grojec et al., 1991; Li et al., 2011).

### Neuropeptide Y and myocardial ischemia/infarction

Animal studies suggest that cardiac NPY is released from sympathetic nerves during experimentally induced myocardial infarction (Han et al., 1989) although its role in this context is still unclear. Several studies have underlined the benefits of the pro-angiogenic properties of NPY in an ischemic environment, including hindlimb ischemia (Lee et al., 2003; Tilan et al., 2013), and chronic myocardial ischemia (Robich et al., 2010; Matyal et al., 2012). Robich et al. demonstrated that 3 weeks of NPY treatment enhanced arteriole formation via the upregulation of DPP-4, Y1R and pro-angiogenic factors including VEGF, endothelial nitric oxide synthase (eNOS) and platelet-derived growth factor (PDGF) in a porcine model of myocardial ischemia (Robich et al., 2010).

Despite these potential beneficial effects, however, other studies have suggested detrimental actions of NPY in relation to myocardial ischemia. A study conducted in dogs found that intra-coronary administration of low dose NPY over 5 min was sufficient to reduce blood flow in the coronary artery by 39%, with unaltered aortic pressure and heart rate (Maturi et al., 1989). The vasoconstrictive effects of NPY in the coronary arteries results in ST-T wave alterations and reduction in intra-myocardial pH and left ventricle ejection fraction, inducing myocardial ischemia in dogs (Maturi et al., 1989). Clarke et al. demonstrated that administration of NPY can induce myocardial ischemia in humans via the initiation of abnormal microvascular vasoconstriction (Clarke et al., 1987). More recently, Rosano et al. (2017) outlined similar effects of NPY-induced transient myocardial ischemia in patients with microvascular angina. This was despite similar vasoconstrictive effects of exogenous-NPY on epicardial coronary artery in patients from both groups. Nonetheless, some argued that the observations obtained in this study could be confounded by the small sample size, gender, age and individual biochemistry profile including blood glucose, LDL and some inflammatory biomarkers (Zhao et al., 2017).

Clinical studies before the advent of percutaneous coronary angiography and modern medical treatment have also shown that peripheral venous “NPY-like activity” is elevated during myocardial infarction and left ventricular failure and correlates with 1-year mortality (Hulting et al., 1990; Ullman et al., 1994a). More recently, we have shown that peripheral venous levels of NPY are significantly elevated in patients undergoing primary percutaneous intervention following ST-elevation myocardial infarction and remain high for at least 48 h (Cuculi et al., 2013). Moreover, venous NPY levels correlated with indices of reperfusion and microvascular resistance. It may be that after placement of a stent in an epicardial coronary artery, local cardiac NPY release limits microvascular flow and leads to larger myocardial infarctions and a worse prognosis. Peripheral venous sampling is likely to be less accurate in determining cardiac NPY release though as hepato-mesenteric release also contributes significantly to circulating levels (Morris et al., 1997). Whilst NPY produces vasoconstriction in canine (Tanaka et al., 1997) and human (Franco-Cereceda et al., 1985; Tseng et al., 1988) epicardial coronary arteries directly and by potentiating norepinephrine mediated vasoconstriction, it has not been directly shown whether NPY vasoconstricts small arteries and arterioles in the coronary microvasculature (which lack alpha_1_ adrenergic receptors).

Overall, NPY therefore seems to play a dual-role in worsening ischemia in the short term but potentially promoting angiogenesis in the longer term. Elucidating the receptor signaling pathways behind these responses therefore warrants further study.

### Neuropeptide Y and cardiac remodeling

Uncontrolled hypertension is a major contributor to the development of more profound cardiovascular diseases, for which initial responses include cardiac remodeling of the left ventricle (left ventricular hypertrophy; LVH) via attenuation of the extracellular matrix and hypertrophic processes (Mcdermott and Bell, 2007). The vasoconstrictive and pro-angiogenic properties of NPY have been found to perform compensatory or detrimental myocardial remodeling in response to hemodynamic overload and ischemia (Zukowska-Grojec et al., 1998a; Ghersi et al., 2001; Mcdermott and Bell, 2007). NPY-receptor specific effects may play a consistent or opposing role on cardiac hypertrophy depending on the circumstances (Erlinge et al., 1994; Zhang et al., 2015b).

Zhang et al. have demonstrated that long-term subcutaneous administration of NPY results in increased systolic blood pressure and hypertension-induced cardiac hypertrophy (Zhang et al., 2015b). Several studies have identified the activation of Y1R and Y2R in ventricular cardiomyocytes to be a consistent player involved in the pathogenesis of cardiac hypertrophy (Nicholl et al., 2002; Allen et al., 2006; Callanan et al., 2007). Y1R and Y2R activity will potentiate elevations in intracellular Ca^2+^/CaMK and MAPK signaling, triggering VSMC mitogenesis and migration and regulating protein turnover and gene expression in hypertrophying cardiomyocytes (Li et al., 2011; Zhang et al., 2015b; Medzikovic et al., 2017). Recent studies have highlighted that NPY-induced Ca^2+^/CaMK and p38 signaling cascades mediate compromised cell viability, energy metabolism and mitochondrial membrane potential integrity in cultured rat cardiomyocytes (Luo et al., 2015; Hu et al., 2017).

Genetic variations in Y2R have been implicated in cardiovascular metabolism, systolic blood pressure variability and LVH (Arnett et al., 2009; Saraf et al., 2016). Y5R synergises with other receptors and has been shown to potentiate MAPK activation (Pellieux et al., 2000) and cardiomyocyte hypertrophic responsiveness in a hypertensive rat model (Bell et al., 2002).

### NPY and ventricular arrhythmias

Increased cardiac sympathetic drive is pro-arrhythmogenic in the context of acute ischemia and chronic heart failure, via increased Ca^2+^ influx (Opie and Clusin, 1990), and this is particularly true in the presence of an already pro-arrhythmogenic substrate (Zipes et al., 2018). However, in addition to the effect of NE there is evidence that NPY released during high level sympathetic stimulation may also increase rates of ventricular arrhythmia. NPY receptors are present on rat and human myocytes (Jonsson-Rylander et al., 2003; Dvorakova et al., 2008), and can affect Ca^2+^ cycling in rat and guinea pig myocytes, although actions do seem to vary depending on species (Millar et al., 1991; Bryant and Hart, 1996; Del Puy Heredia et al., 2005). Furthermore, NE lowers the ventricular fibrillation threshold (VFT) in a Langendorff perfused rat heart model with intact innervation; an effect blocked by metoprolol as expected. However, metoprolol does not have this effect in the presence of high frequency stellate ganglion stimulation (Herring, 2015). This may be caused by the co-release of NPY, as exogenous NPY also lowers VFT, and lowered VFT due to stellate stimulation is abrogated by the combined use of Metoprolol and an NPY antagonist.

A direct action on cardiomyocytes does not appear to be the only mechanism by which NPY could be pro-arrhythmogenic. Inhibition of vagally mediated bradycardia by sympathetic nerve stimulation was first found to be potentially mediated by NPY by Potter (Potter, 1985), and was subsequently shown to be mediated by the Y2R through the use of a Y2R antagonist (Smith-White et al., 2001; Herring et al., 2012) and Y2 receptor knock-out (Smith-White et al., 2002). Y2Rs are found on cholinergic vagal neurons in the guinea pig right atrium and around the sino-atrial node, and exogenous NPY limits bradycardia in response to vagus nerve stimulation but not an acetylcholine analog (Herring et al., 2008). This appears to be via a Y2R and PKC-dependent mechanism reducing acetylcholine release and provides evidence of sympatho-vagal cross talk. The protective effect of vagal stimulation (Myers et al., 1974; Nash et al., 2001; Ng et al., 2007; Kalla et al., 2016) may therefore be limited by the co-release of NPY during high-level sympathetic activation. However, in a rat model of acute myocardial infarction, use of an Y2R antagonist did not reduce rates of ventricular arrhythmia compared to placebo (Omerovic et al., 2007). Similarly, rates of ventricular arrhythmia in a porcine ischemia-reperfusion model were not significantly different in animals infused with a Y2R antagonist (Ilebekk et al., 2006). However, the efficacy of Y2R blockade is likely to depend on the size of infarction and subsequent level of sympathetic hyperactivity and NPY release. Certainly in a clinical ST elevation myocardial infarction (STEMI) population who underwent coronary revascularization, high NPY levels appeared to be correlated with increased severity of ventricular arrhythmias during inpatient admission, despite similar left ventricular ejection fractions, troponin levels and beta-blocker usage (Herring et al., 2016). This suggests that NPY may have an important role in the pathogenesis of post-myocardial infarction arrhythmias in a clinical population, and is an area which warrants further investigation.

### NPY and heart failure

Systolic heart failure is associated with increased sympathetic nervous system activity and reduction in parasympathetic activity (Oberhauser et al., 2001; Sheng and Zhu, 2018). Early studies showed high baseline levels in heart failure patients (Maisel et al., 1989; Hulting et al., 1990; Madsen et al., 1993; Ullman et al., 1994b; Schwertfeger et al., 2004), and indeed there is evidence that severity of heart failure may be correlated to NPY level (Liu et al., 1994). The ability to further increase NPY release in patients with heart failure, may be related to the severity of the condition. In two studies which measured NPY levels in heart failure patients post-exercise, one found no significant increase, whilst one found an increase from 50+/−5 to 60+/−6 pmol/l (Maisel et al., 1989; Ullman et al., 1994a). A more recent study has also confirmed higher NPY levels in heart failure patients, which correlated with BNP levels and echocardiographic markers of heart failure severity (Ozkaramanli Gur et al., 2017).

In a study of 9 patients admitted with acute heart failure, elevated catecholamine and NPY levels were only observed in the single patient who did not improve with treatment and subsequently died (Missouris et al., 1998). Other small studies have only found a trend for elevated NPY levels for example in heart failure patients undergoing invasive haemodynamic studies in response to dobutamine (Dubois-Rande et al., 1992), and in response to exercise (Nicholls et al., 1992). It has been postulated that these differences may be due to high NPY levels only occurring when there is sustained and sufficiently high sympathetic activation (Pedrazzini et al., 2003). As recently discussed (Widiapradja et al., 2017), it should be noted that there may also be differences between the plasma levels of NPY measured in these studies, and actual cardiac tissue concentrations. A high cardiac output model of heart failure in the rat found high plasma levels but reduced tissue levels in the heart (Callanan et al., 2007) although this may be as a result of increased cardiac release of NPY. As well as changes in NPY levels, the same study found a shift in mRNA from the Y1R to the Y2R in heart failure, and this may represent another way in which differential effects are mediated. This has also been shown in humans, with lower levels of Y1R mRNA in endomyocardial biopsies from end-stage heart failure hearts at the time of transplant and in transplanted hearts 1 year post-transplant, compared to control donor hearts prior to transplant (Gullestad et al., 1998). It should, however, be noted that donors were younger than the two patient groups in this case. Increased NPY levels have also been specifically found in a rat model of stress cardiomyopathy (Ananda et al., 2012), as well as in a case report of a patient with stress cardiomyopathy (Szardien et al., 2011).

Different mechanisms that may mediate the association of dysregulated NPY signaling and heart failure have been suggested. NPY has been found to damage mitochondrial structure and decrease energy metabolism in neonatal rat cardiomyocytes (Luo et al., 2015). In addition, there is evidence that NPY activates rat cardiac fibroblasts (Zhu et al., 2015), an effect blocked by Y1R antagonist, and cardiac fibroblasts are known to be key to cardiac remodeling and fibrosis and are thus central to the pathogenesis of heart failure (Brown et al., 2005).

In spite of this, increased NPY levels may also play a protective role in heart failure. NPY has been shown to have diuretic and natriuretic actions in the kidneys, despite reduced renal blood blow (Allen et al., 1985). There is also evidence that it can reduce renin levels in rat models (Pfister et al., 1986), including in a rat model of heart failure (Zelis et al., 1994).

### Neuropeptide Y and diabetic cardiomyopathy

Diabetic cardiomyopathy is a recognized entity in diabetic patients without overt coronary disease (Giles and Sander, 2004). Given the autonomic neuropathy associated with diabetes, it has been postulated that diabetic cardiomyopathy may be caused by dysregulated neuropeptide signaling and imbalance of sympathetic-parasympathetic crosstalk in the myocardium as a result of autonomic neuropathy (Ejaz et al., 2011). This includes NPY, and indeed reduced NPY levels in response to hypoglycaemia have been found in patients with autonomic neuropathy as opposed to without (Bolinder et al., 2002). It has even been suggested that basal NPY levels may be predictive of sympathetic nerve failure in patients with autonomic neuropathy (Sundkvist et al., 1992). Although plasma levels of NPY have been found to be higher in patients with chronic type 2 diabetes (Matyal et al., 2011), it has been postulated that this may be due to a compensatory increase in extra-neuronal NPY (Ejaz et al., 2011), and in the same study right atrial NPY mRNA expression was reduced compared to non-diabetic patients, along with an increase in Y2R and Y5R.

### Neuropeptide Y and genetic polymorphism

Genetic polymorphism in NPY receptors (Y1, Y2, and Y5) have been associated with cardiovascular dysfunction and predisposal to early on-set cardiovascular risk (Arnett et al., 2009; Shah et al., 2009). A common leucine7-to-proline7 polymorphism in the single peptide of the NPY gene was found to be associated with increased stress-induced plasma NPY levels, hyperlipidaemia, compromised low-density lipoprotein cholesterol metabolism as well as accelerated atherosclerosis, hypertension and coronary heart disease (Niskanen et al., 2000; Karvonen et al., 2001). Different NPY single nucleotide polymorphisms have also been linked to higher plasma NPY levels and are reported to predict early onset atherosclerosis in US populations (Shah et al., 2009). In addition, a recent study by Chang et al. observed an altered cardiac vagal outflow and perceived stress with a NPY promoter polymorphism (rs16147), suggesting a potential parasympathetic role for NPY in the modulation of stress (Chang et al., 2016).

## Future directions

The wide range of involvement of NPY in cardiovascular disease processes makes further study into its associations and actions an exciting prospect. An initial way in which it may be incorporated into clinical practice is as a marker of increased sympathetic activity, and so clinical risk. For example, its apparent correlation with post-myocardial ventricular arrhythmias (Herring et al., 2016) could allow it to eventually be used to aid risk stratification in this population.

As the specific roles of NPY in each disease process are further elucidated, this will hopefully lead to therapeutic pharmacological targeting of specific NPY receptors. Use of NPY receptor antagonists could have significant future clinical use, e.g., in the treatment of arrhythmias and ischemia. However, further pre-clinical studies need to be done to clarify the involvement of NPY, and the effects of specific antagonists, in order to make this into a possibility.

## Conclusion

Since its discovery in 1982, NPY has increasingly been found to play a role in a wide range of physiological functions, across a number of organ systems. In the cardiovascular system, it has effects on blood pressure, vasoconstriction, atherosclerotic plaque formation, arrhythmia and angiogenesis, amongst others. It therefore has the potential to be a key player in a number of cardiovascular disease processes. However, the range of its effects makes defining a predominantly cardio-protective or pathogenic role extremely difficult. Indeed, it has been postulated that its overall effect may be dose dependent, with protective effects at low doses and pathogenic effects at higher doses. The exact time point in the disease process may be therefore be key to understanding its regulatory role. In addition to this, altered NPY receptor sub-type expression has the ability to change the overall influence of released NPY, again modulating its effects. Studies may be further complicated by how NPY levels are measured, as plasma and local tissue concentrations are likely to differ. The high burden of cardiovascular disease, combined with the pleiotropic influence of NPY, demonstrates the importance of further elucidating the role of NPY in cardiovascular disease processes. The potential to target NPY and its receptors to modify these processes is an important area for future research.

## Author contributions

NH conceived the manuscript. CT and PG produced the first draft and all authors edited and agreed to submission.

### Conflict of interest statement

The authors declare that the research was conducted in the absence of any commercial or financial relationships that could be construed as a potential conflict of interest.
